# When “good” is not always right: effect of the consequences of motor action on valence-space associations

**DOI:** 10.3389/fpsyg.2015.00237

**Published:** 2015-03-05

**Authors:** Denis Brouillet, Audrey Milhau, Thibaut Brouillet

**Affiliations:** ^1^EPSYLON Laboratory, Paul Valery UniversityMontpellier, France; ^2^URECA Laboratory, Lille 3 UniversityLille, France; ^3^CERSÆM Laboratory, Université Paris Ouest-La DéfenseNanterre, France

**Keywords:** *Body-Specificity Hypothesis*, motor fluency, valence-laterality associations, event coding, consequences of actions

## Abstract

Since the work of Casasanto (2009), it is now well established that valence and laterality are associated. Participants tend to prefer objects presented on their dominant side over items presented on their non-dominant side, and to place good items on their dominant side and bad items on the other side. Several studies highlight that those associations of valence and laterality are accounted for by the greater motor fluency of the dominant hand and various studies noted that these associations could be reversed depending on the way people interact with their environment. Consistently with the Theory of Event Coding, the aim of this work is to show that the consequences of motor actions could also reverse the associations between valence and laterality. Thus, if participants had to place two animals (one good, one bad) on two supports, one stable (no risk of falling), one unstable (risk of falling), we hypothesized that the good item would be placed on the stable support, regardless of the side where it would be put (i.e., on the dominant or non-dominant side). We expected the opposite for the bad item. The results of two experiments are consistent with this prediction and support the claim that the consequences of motor action bias the hedonic connotation of our dominant side.

## Introduction

In various languages and cultures, phrases and idioms express a link between valence and horizontal space: to be someone's right hand, to have two left feet… In all these expressions, good things tend to be associated with the right side and bad things with the left side.

According to Casasanto's *Body-Specificity Hypothesis* (2009, 2011), the way we interact with our environment participates in our conceptualization of concepts and meaning. For instance, valence is associated with horizontal space because of the motor fluency by which one acts with one's dominant hand (i.e., the association valence-laterality). Indeed, one of our main body specificities is our handedness. Right- and left-handers act differently in their environment, and experience fluency from opposed movements: our most fluent actions are those carried out by our dominant hand, and on our dominant side.

Various researches have shown that motor fluency is associated with a hedonic connotation, such as fluent actions are positively connoted compared with a less or non-fluent action (Reber et al., 1998; Winkielman and Cacioppo, 2001; Winkielman et al., 2003; Hayes et al., 2008; Brouillet et al., 2011; de la Vega et al., 2012).

However Casasanto and Chrysikou (2011, Experiment 2) highlighted that the association valence-laterality could be reversed by short-term changes in the way one interact with its environment (i.e., participants manipulated dominos while wearing a bulky ski glove on their dominant hand). The aim of this work is to show that the consequences of motor actions could also reverse the associations between valence and laterality.

Indeed, since James's (1890) ideomotor theory, it is known that learning establishes direct and automatic links between actions and the perceptual results they generate (for a review, see Stock and Stock, 2004; Shin et al., 2010). In other words, action execution is triggered by a stimulus, and is necessarily followed by a feedback, which is a function of this action (action effect, Hommel, 1996, 2013; Prinz, 1997). In line with the ideomotor theory (James, 1890), the Theory of Event Coding (i.e., TEC, Prinz, 1997; Hommel et al., 2001) considers that action and its effects constitute one and the same event. In this line, Brouillet et al. (2014) could show that sensory-motor consequences of past actions form part of the memory trace components cued by a current action.

The purpose of this research is to test the hypothesis that the hedonic connotation of the action performed with our dominant hand on our dominant side (i.e., motor fluency) is relative to the consequences of this action. If the consequences of this action are detrimental to the object be placed (e.g., fall risk), we can assume that the association “right space—positive valence” will not occur.

### Valence and laterality

Casasanto (2009) conducted the first study that directly tested the associations on valence and laterality. In their first experiment, participants were presented with a character named Bob that was supposed to like pandas and thought they were good, and to dislike zebras, thinking they were bad. Participants had to place those two animals into two boxes presented on the left and on the right parts of a piece of paper. The main result obtained by Casasanto is that right-handers tended to place the good panda in the right box, and the bad zebra in the left box, while a majority of left-handers placed the good panda on the left box, and the bad zebra on the right one (Casasanto, 2009, Experiment 1). Identical results were found when Bob was supposed to like zebras, and to dislike pandas. In following experiments, participants tended to prefer the product, person or creature presented on their dominant side to the items presented on their non-dominant side (Casasanto, 2009, Experiments 3–5). For example when facing two alien creatures placed on either side of a piece of paper and having to choose which of them looks the most intelligent, funny or honest, participants tended to choose the one situated on their dominant side (Casasanto, 2009, Experiment 4). Simple observations of hand gestures during speech highlight similar combinations of valence and side, depending on the orator's handedness: during the campaigns for the U.S. presidential election, Casasanto and Jasmin (2010) identified different gestures during positive and negative speeches, depending on the candidates' dominant hand: right-handed candidates (Bush and Kerry) used their right hand during positively connoted speeches, and their left-hand during negatively connoted discourses, while left-handed candidates (Obama and McCain) used their left hand for positive speeches and their right-hand for negative ones.

These spontaneous associations of positive valence with the dominant side seem to be rather robust since they have been demonstrated in children as young as 5 years old (Casasanto and Henetz, 2012) and in various cultures (de la Fuente et al., 2014). Furthermore, they affect people's memory as well as their judgments: Brunyé et al. (2012) showed participants a map featuring the positions of fictitious positive and negative events. Later, participants had to recall those locations on the map. Results demonstrated memory biases dependent on the valence of the event and on the handedness of the participants: right-handers tended to locate positive events too far to the right and negative events too far to the left, whereas left-handers showed opposite biases.

These studies focused on the spontaneous associations of valence and laterality. Recently, the question was raised of whether those associations could affect response times in a valence judgment task manipulating the compatibility between valence and response hand. de la Vega et al. (2012) tested right-handers and left-handers using a valence judgment task of emotional words. Participants were asked to respond with both hands (i.e., dominant and non-dominant) by pressing response keys located respectively on the left and on the right-hand side of a keyboard, corresponding to either a positive or negative response (the position of each valence was reversed in the second part of the experiment). Their results showed compatibility effects: both right- and left-handers responded faster with their dominant hand to positive words than to negative words (see Kong, 2013, for similar results on evaluation of faces).

One experiment has focused on the effect of the lateral position of the positive and negative responses on the evaluation of neutral words. Milhau et al. (2013) showed that in a valence judgment task the way positive and negative labels are presented on each side of a horizontal scale has an impact on judgment: right-handers' evaluations are more positive on a scale associating positive to the right and negative to the left (congruently with Casasanto's associations of valence and laterality in right-handers) than on the reversed scale, especially after the carrying out of a fluent movement of the right hand.

Since those associations of valence and laterality are explained on the basis of manual dominance, one could expect that these links are fixed and constant. Yet, Casasanto and Chrysikou (2011) noted that these associations could be reversed by both long-term and short-term changes in the way one interacts with its environment. In a first experiment, stroke-induced hemiplegic patients were asked to perform an oral version of the good/bad animals experiment conducted by Casasanto (2009). In the second experiment, the authors used a simple motor task requiring the two hands (manipulating dominos), and temporarily disabled right-handers and left-handers' dominant hand with a bulky ski glove, leaving their non-dominant hand more efficient for task performance. Results showed that right-handers paralyzed (Experiment 1) or virtually disabled (Experiment 2) on the right side tended to manifest valence/laterality associations usually encountered in left-handers, specifically to associate positive with the left and negative with the right. Similarly, left-handed patients paralyzed/disabled on the left side manifested right-handers' valence/laterality mapping: positive on the right and negative on the left (Casasanto and Chrysikou, 2011). These results confirmed that modifications in the way people interact with their environment (even at very short-term) modify their motor fluency and therefore their valence/laterality associations.

Similarly, Milhau et al. (2014) showed that in a valence judgment task, right-handers and left-handers manifest the same pattern of compatibility effects when using the same hand of response. In a valence judgment task of positive and negative words, participants responded with lateralized actions of either their dominant or non-dominant hand. Results highlighted that for both right- and left-handers, when the location of responses was congruent with the fluency of the responding hand (for the right hand: negative/left and positive/right; for the left hand: positive/left and negative/right), response times to positive evaluations were shorter than for negative evaluations. Conversely, when the location of responses was non-congruent with the fluency of the responding hand, we observed faster responses for negative evaluations than for positive evaluations.

These studies support an account of the associations of valence and laterality based on motor fluency and not only in terms of handedness. It is not always the dominant side that is positively connoted, but the side of the most fluent action. A recent experiment by de la Vega et al. (2013) confirms this explanation, demonstrating that the compatibility effects of valence and laterality in a valence judgment task are based on the response hand, and not on the response side: dominant-hand responses are facilitated for positive evaluations, even when the responses are located on the non-dominant side.

From these last experiments motor fluency seems to be the key factor to explain hedonic connotation linked to action. However, it appears that all these studies overlooked a crucial aspect of action: each action is followed by consequences. The status of action in these experiments is only a response to a stimulus, the command you run to respond to the task. Yet, in real life, an action is not only an output of the system, it is also informative of the consequences associated for the person performing it (see above ideomotor theory and TEC). For example, Anelli et al. (2012) show that if generally graspable objects activate a facilitating motor response, dangerous objects do not. Our objective is therefore to take into account the impact of action's consequences on the link between horizontal space and valence.

## Experiment 1

### Overview

Our objective in this experiment was to determine whether associations between valence and horizontal space in right-handed participants are influenced by the consequences of the participants' actions.

We first intended to replicate Casasanto's (2009) classic result that right-handers tend to associate good with right and bad with left (Experiment 1a). Then we tested whether these classical associations would appear with our specific response device (Experiment 1b). Finally, our last and main objective was to determine the impact of action consequences on these associations, by manipulating the risk associated with the result of an action (Experiments 1c and 1d).

In four declinations of the experiment, participants were presented with two figurines of animals (one presented as good and the other as bad, see Supplementary Material), and were instructed to place, with their right hand, each of them on one of two small planks situated on the participant's left and right-hand side (see Supplementary Material). In Experiment 1a, the two small planks were laid flat on the table. In Experiment 1b, the two small planks were placed in stable equilibrium on a wood lath. In Experiment 1c, the small plank on the right side was placed precariously on a wood lath and was in unstable equilibrium, while the plank on the left side stood stable. In Experiment 1d, the small plank on the left side was placed precariously on a wood lath and was in instable equilibrium, while the plank on the right side stood stable. Thus, in Experiments 1c and 1d putting the animal on the instable small plank would result in the animal falling.

## Experiment 1a

### Participants

Thirty two students from Montpellier University (25 females), all native French speakers and all right-handed, took part in this experiment after having signed informed consent to participate in the study.

### Material

The material comprised two plastic colored animals and two small planks.

The plastic animals were a hyena (8 cm long and 4 cm high) and a zebra (8 cm long and 6 cm high). We chose these two plastic animals because they were equally long (this feature is important for the position of each animal on the small plank). A black mark, like an inverted triangle, was painted on the belly of the animal that referred to the middle. A pre-test on 50 persons determined that the hyena was negatively valenced and the zebra positively valenced.

The two small planks were 15 cm long, 3 cm wide and 1 cm deep. A white point with a diameter of 0.5 cm was painted on the middle of the long face of each small plank. This point indicated where participants had to put each animal.

### Procedure

Participants sat at a table with the experimenter sitting in front of them. A box containing the material was placed on a chair next to the experimenter, so that participants could not see the material before the experimenter presented them with it. A sheet of stiff paper was placed on the table (80 cm long and 50 cm wide), the bottom edge aligned with the edge of the table. Two crosses were horizontally aligned and drawn 50 cm apart on the paper sheet. Each cross was positioned 15 cm from the right or left edge of the sheet. This device allowed for similar conditions of presentation among participants.

Each participant was tested individually in an isolated room by a single experimenter. Each experiment started with a brief presentation of the upcoming task; participants indicated whether they agreed to participate or not. Upon their approval, the experiment started.

The experiment consisted in only one trial. The experimenter took out the two animals in one hand. He asked the participants to reach out their left hand and deposited the two animals together. He asked them to put their hands onto their knees until further instructions. This procedure was meant to avoid presenting an animal before the other, thus inducing a preferential choice. Once the participant's hand was on his knees, the experimenter took out the wooden planks and placed them one on each cross. Participants were then asked to place, with their right hand, the good zebra on one of the two small planks laid on the table and the bad hyena on the other by matching the mark on the animal's belly with the white point on the small plank. As in Casasanto (2009), the order in which participants were instructed to place the good and bad animals was counterbalanced, to ensure that any associations between space and valence in participants' judgments were not confounded with associations between the side of space and the temporal order in which they placed the animals (16 participants were instructed for good animal first and 16 for bad animal first).

### Results and discussion

**Figure 1:**
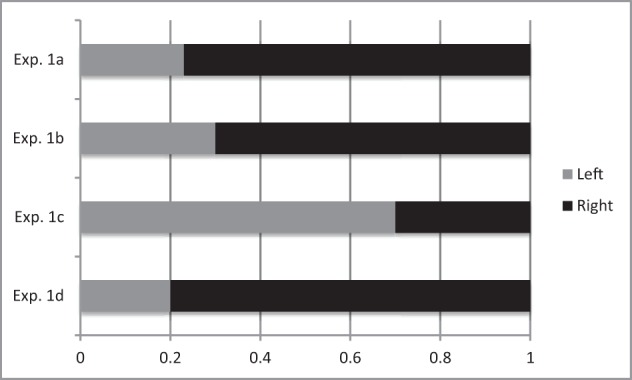
**Proportion of participants who placed the good animal on the small planks on the left and on the right**. Note that in Experiment 1c the animal placed on the plank on the right hand side fell, while in Experiment 1d the animal placed on the plank on the left hand side fell. In Experiments 1a and 1b the animals could not fall.

When asked to freely place a good animal and a bad animal on their left and right side, a majority (78%) of our right-handed participants placed the good animal on the small plank on the right, and the bad one on the small plank on the left (sign test on 25 vs. 7, *Z* = 4.00, *p* < 0.01, see Figure 1).

These results replicate Casasanto's (2009) classic effect that people tend to place positive items on their dominant side, and bad items on their non-dominant side.

Our effect is consistent with the Good is Right association usually found in right-handers (Casasanto, 2009; de la Vega et al., 2012; Milhau et al., 2013, 2014). The following experiment will try to extend this effect to our specific device.

## Experiment 1b

### Participants

Thirty two students from Montpellier University (23 females) that did not participate in Experiment 1a took part in this experiment. They were all native French speakers and all right-handed, and signed informed consent to participate in the study.

### Material

The material was the same as in Experiment 1a, except that the small planks had a black mark, like an inverted triangle, painted in the middle of the edge of the plank. We also used two wood laths that were 10 cm long, 3 cm large and 3 cm deep. A black triangle was painted at the end of the wood laths, on their edges.

### Procedure

The procedure was very similar to Experiment 1a, except that here the experimenter first took out the two wood laths and placed then vertically on the crosses on the table. Then the experimenter took out one center-marked small plank and asked participants to put it on the wood lath on the right by matching the mark of the small plank with the mark of the wood lath. When the small plank was placed the experimenter took out the other center-marked small plank and asked participants to put it on the wood lath on the left by matching the mark of the small plank with the mark of the wood lath. After these manipulations the two small planks were in stable equilibrium on the two wood laths and did not present any risk. Participants were then asked to place, with their right hand, the two animals on each of the two small planks. The order in which participants were instructed to place the small planks and the two animals was counterbalanced.

### Results and discussion

When asked to freely place a good animal and a bad animal on their left and right sides on stable planks, a majority (69%) of our right-handed participants placed the good animal on the small plank on the right, and the bad one on the small plank on the left (sign test on 22 vs. 10, *Z* = 3.17, *p* < 0.01, see Figure 1).

These results replicate both Casasanto's effect (2009) and our results in Experiment 1a.

It confirms that our device with planks in equilibrium is adequate to further explore the associations of valence and laterality.

Our objective in Experiments 1c and 1d is now to explore the impact of action consequences on these associations.

## Experiment 1c

### Participants

Thirty two students from Montpellier University (26 females) that did not participate in Experiments 1a and 1b took part in this experiment. They were all native French speakers and all right-handed, and signed informed consent to participate in the study.

### Material

The material was the same as in Experiment 1b, except that one small plank had a black mark painted on the middle of the edge of the plank (center-marked) while the other plank had a black mark painted at 5 cm from the left edge of the plank (left-marked). We also used two wood laths that were 10 cm long, 3 cm large, and 3 cm deep. A black triangle was painted at the end of the wood laths, on the edge.

### Procedure

The procedure was very similar from Experiment 1b, except that here participant were instructed to place the left-marked plank on the wood lath on the right, matching the mark on the small plank with the mark on the wood lath. The matching of the marks resulted in the small plank being in instable equilibrium on the wood lath. Note that by placing the small plank participants experienced the instability and therefore the fact that an object placed on it would fall systematically from the structure. When the small plank was placed, the experimenter took out the center-marked small plank and asked participants to put it on the wood lath on the left by matching the mark of the small plank with the mark of the wood lath. In this case the small plank was in stable equilibrium on the wood lath. Participants had to place, with their right hand, one animal on each plank.

### Results and discussion

Contrary to our previous results and consistently with our predictions, a majority (69%) of our right-handed participants chose to place the good animal on the small plank on the left and the bad animal on the plank on the right (sign test on 22 vs. 10, *Z* = 3.17, *p* < 0.01 (see Figure 1).

This result indicates an inversion of right-handers' usual associations, and highlights that participants took into account the consequences of their action. Because the animal placed on the unstable plank on the right would fall, participants mostly chose not to place the good animal on it, and to place it on the stable (“safe”) plank, rather, even if it was on the left side.

## Experiment 1d

### Participants

Thirty-two students from Montpellier University (25 females) that did not participate in previous experiments took part in this experiment. They were all native French speakers and all right-handed, and signed informed consent to participate in the study.

### Material

The material was the same as in Experiment 1c, except that one small plank had a black mark painted in the middle of the edge of the plank (center-marked) while the other plank had a black mark painted 5 cm from the right edge of the plank (right-marked). We also used two wood laths that were 10 cm long, 3 cm large, and 3 cm deep. A black triangle was painted at the end of the wood laths, on their edges.

### Procedure

The procedure was very similar from Experiment 1c, except that here the unstable equilibrium plank was on the wood lath on the left, and the stable plank was on the wood lath on the right. Participants had to place, with their right hand, one animal on each plank. Note that the animal placed on the plank on the left hand side fell systematically from the structure.

### Results and discussion

The pattern of result in this fourth experiment is in line with those of Experiments 1a and 1b and is the exact opposite of the pattern in Experiment 1d. A majority (81%) of our right-handed participants chose to place the good animal on the small plank on the right and the bad animal on the plank on the left (sign test on 26 vs. 6, *Z* = 4.24, *p* < 0.01, see Figure 1).

When it was the plank on the left that fell, participants had no problem assigning it to the bad animal. Their choices consequently reflect their typical associations of valence and laterality, linking good with right and bad with left.

## Conclusion of experiment 1

The aim of this experiment was to test associations between valence and horizontal space in right-handed participants to determine whether these associations were influenced by the consequences of participants' actions.

Usually right-handers tend to associate positive to their right part of space and negative to their left part (Casasanto, 2009; de la Vega et al., 2012), because of the greater ease of their interaction with the word with their dominant right hand (motor fluency, for extended explanation see Milhau et al., 2014).

Experiment 1a allowed us to replicate these associations when we manipulated only the animal affective trait and the position of the planks. In this situation the action of placing each animal on the plank did not have any overt consequence, and right-handers mostly placed the good animal on the plank on the right and the bad animal on the plank on the left (see Figure 1).

In Experiment 1b the consequences of the participants' actions were limited: placing the animal on the planks was not likely to make it fall, whether it was placed on the right-hand-side plank or on the left-hand-side plank. This situation therefore did not differ significantly from the one in Experiment 1a, and we observed a similar pattern of response: good is right.

Our main hypothesis was that an overt “bad” consequence of action would impact participants' choices in this task. In Experiments 1c and 1d we modified the task so that one of the response choices would induce a risk for the animal: by making one of the planks unstable, one of the animals fell from the plank when the participant put it on it.

Our main result in Experiment 1 is the demonstration of the inversion of responses in Experiment 1c. When the unstable small plank was on the right side, participants did not choose to place the good animal on it as right-handers had done in the previous experiments. In contrast they largely preferred to place the bad animal on it, as if they were trying to “protect” the good animal from the risk of falling.

Experiment 1d confirmed this interpretation since when the unstable plank was on the left, right-handers mostly chose to place the bad animal on it, manifesting in their turn the usual associations according to which good is right, bad is left.

To test the validity of our interpretation in terms of “trying to protect the good animal from the risk of falling”, we conducted a second experiment in which in a first phase the action of placing the good animal on the plank did not have overt consequence (i.e., similar to Experiment 1a), but in a second phase the action of placing the animal could have dramatic consequences (i.e., similar to Experiment 1c). In other words we wanted to see if after placing the good animal on the right, participants were able to change their choice when their action could have bad consequences for it (i.e., the risk of falling).

## Experiment 2

Experiment 2 was composed of two declinations that differed only in the second phase. In the shared first phase the two small planks were laid on the table, one on the right side and one on the left side of the participants. During the second phase the small planks were placed on the top of a wood lath. In Experiment 2a one of the small plank was in stable equilibrium on the wood lath on the right and the other in instable equilibrium on the wood lath on the left. In Experiment 2b one of the small planks was in instable equilibrium on the wood lath on the right and the other in stable equilibrium on the wood lath on the left.

## Experiment 2a

### Participants

Thirty-two students from Montpellier University (22 females), all native French speakers and all right-handed, took part in this experiment after having signed informed consent to participate in the study.

### Material

The material was the same as in Experiment 1a and in Experiment 1c.

### Procedure

Participants were not informed that they would have to place animals on the two small planks two times in a row. The procedure for the first placement was very similar from Experiment 1a, with the two small planks posed directly on the table. The procedure for the second placement was very similar from Experiment 1d: the two small planks were in equilibrium on the wood lath the one on the right being stable and the one on the left being unstable.

### Results and discussion

**Figure 2:**
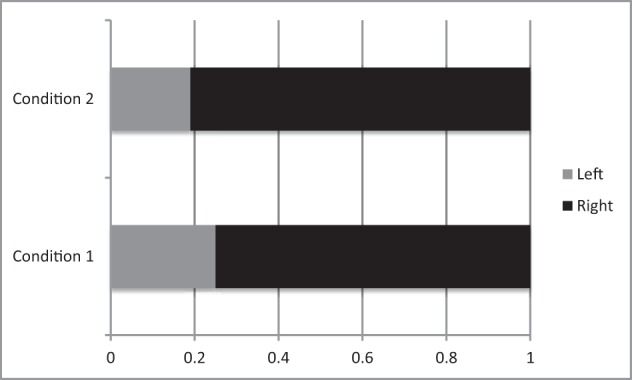
**Proportion of participants who placed the good animal on the planks on the left and on the right**. Note that in condition 1 the animals could not fall but in condition 2 the animal placed on the left plank fell.

The results of the first placement show that a majority (75%) of our right-handed participants placed the good animal on the small plank on the right, and the bad one on the small plank on the left (sign test on 24 vs. 8, *Z* = 3.75, *p* < 0.01, see Figure 2).

The results of the second placement show that a majority (81%) of our right-handed participants chose to place the good animal on the small plank on the right and the bad animal on the plank on the left (sign test on 26 vs. 6, *Z* = 4.24, *p* < 0.01, see Figure 2).

The results are consistent with those obtained in Experiments 1a and 1d. When asked to freely place a good animal and a bad animal on their left and right sides, participants placed the good animal on the small plank on the right, and the bad one on the small plank on the left. Their choices were similar in both phases, showing that the risk of falling associated to the left plank did not impact their responses.

## Experiment 2b

### Participants

Thirty-two students from Montpellier University (26 females), all native French speakers and all right-handed, took part in this experiment after having signed informed consent to participate in the study.

### Material

The material was the same as in Experiment 2a.

### Procedure

Participants were not informed that they would have to place animals on the two small planks two times in a row. The procedure for the first placement was very similar to that in Experiment 1a, with the two small planks laid directly on the table. The procedure for the second placement was very similar from Experiment 1c: the two small planks were in equilibrium on the wood lath the one on the right being unstable and the one on the left being stable.

### Results and discussion

**Figure 3:**
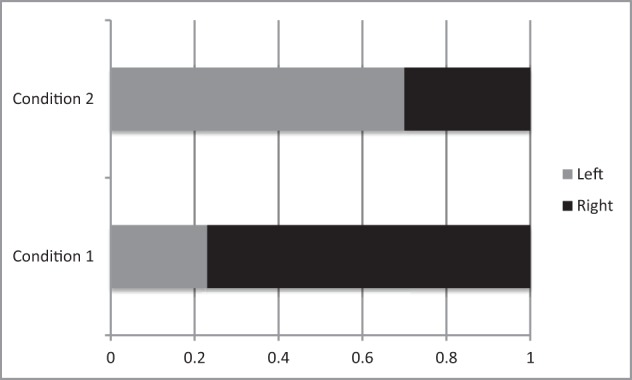
**Proportion of participants who placed the good animal on the small planks on the left and on the right**. Note that in condition 1 the animals could not fall but in condition 2 the animal placed on the right plank fell.

The results of the first placement show that a majority (78%) of our right-handed participants placed the good animal on the small plank on the right, and the bad one on the small plank on the left (sign test on 25 vs. 7, *Z* = 4.00, *p* < 0.01, see Figure 3).

The results of the second placement show that a majority (72%) of our right-handed participants chose to place the good animal on the small plank on the left and the bad animal on the plank on the right (sign test on 23 vs. 9, *Z* = 3.47, *p* < 0.01, see Figure 3).

The results are consistent with those obtained in Experiments 1a and 1c. When asked to freely place a good animal and a bad animal on safe small planks laid on their left and right sides, participants placed the good animal on the small plank on the right, and the bad one on the small plank on the left. But when the small plank on the right would fall, participants reversed their previous placement, and chose not to place the good animal on it but rather to place it on the safety plank (stable), even if it was on the left side.

## Conclusion of experiment 2

The aim of Experiment 1 was to test whether the associations between valence and horizontal space in right-handed participants, are influenced by the consequences of participants' actions. Results of Experiment 1c highlight that when the unstable plank was on the right side, participants preferred to place the bad animal on it, as if they were trying to “protect” the good animal from the risk of falling.

The aim of this Experiment 2 was to test this interpretation. The results highlight that, even if in a first phase participants placed spontaneously the good animal on the right side, when the unstable plank was on the right side, participants did not choose to place the good animal on it. In contrast they largely preferred to place it on the left side. These results support our hypothesis that an overt “bad” consequence of action would impact participants' choices.

## General discussion

The *Body-Specificity Hypothesis* (Casasanto, 2009, 2011) suggests that the body and the way one uses it shape one's thoughts. This proposition has been tested by comparing right- and left-handers' emotional judgment. Because of handedness, people experience horizontal space differently: they act more easily with their dominant hand, that is to say more fluently. Since motor fluency is associated with hedonic connotation, a fluent movement being ascribed a positive connotation (Hayes et al., 2008), people tend to associate positive valence with the dominant side: right is good and left is bad for a right-hander, the reverse for a left-hander (Casasanto and Henetz, 2012; de la Vega et al., 2012, 2013; Kong, 2013; de la Fuente et al., 2014; Milhau et al., 2014). Nonetheless Casasanto and Chrysikou (2011) showed that variations in the way people interact with their environment modify their valence—laterality associations. Moreover, Milhau et al. (2013) showed that the location of responses interacts with the fluency of the responding hand. Thus, these studies highlight that the valence-laterality association is supported by motor fluency more than handedness. It is not always the dominant side that is positively connoted, but the side of the most fluent action.

Here we considered that these last studies overlooked a crucial aspect of action: each action is followed by consequences. As supported by the ideomotor theory (James, 1890) or the Theory of Event Coding (Prinz, 1997; Hommel et al., 2001), action and its effects constitute one and the same event. Therefore, the aim of this article was to take into account the impact of action's consequences on the link between valence and laterality.

Our experiments are inspired by Casasanto's (2009) classic paradigm. Participants were presented two figurines of animals (one presented as good and the other as bad), and were instructed to place each of them on one of two small planks situated on the participant's left and right-hand side. These small planks were either laid flat on the table or the participants had first to place them on wood laths. In this case the small planks were in stable or unstable equilibrium.

The main result in Experiment 1 is that when the unstable small plank was on the right side, participants did not choose to place the good animal on it. In contrast they largely preferred to place the good animal on the left side on the stable small plank, as if they were trying to protect the good animal from the risk of falling.

The results of Experiment 2 highlight that, even if the participants spontaneously placed the good animal on the right when the device was safe, they changed their responses when it was made dangerous (i.e., risk of falling): they did not put the good animal on the risky right side but preferred to put it on the left side, in a safer place.

Taken together, these results support our hypothesis that the hedonic connotation associated to the fluency linked to dominant hand and dominant side is relative to the consequences of the actions performed. The classic association “right space—positive valence” for a right-hander did not occur if the action performed could have negative consequences (e.g., fall risk). In other words, right is good if right is safe. But, the experience should be replicated with left-handers for the results to be generalizable.

To conclude, the originality of this work lies in three points. First, it is to our knowledge the first study that shows that the consequences of an action modify emotional judgments and reverse the valence—space associations. This result provides additional support to the claim that the compatibility effects of valence and laterality in a valence judgment task are based on the fluency of the response hand, and not on the response side: this is true despite the consequences of the action performed. Second, the perception of a situation is underpinned by the activation of the perceptual outcome of action, that is to say the action performed and its consequences. Consequently, the judgment on an object that appears in this situation and the action to perform with it are dependent on this perceptual outcome. Third, we have created an original paradigm that simulates what happens in real life: actions are always followed by an informative feedback. Indeed, the functional characteristics are crucial to define a fluent action (i.e., effector and orientation, for example the dominant hand acting in the dominant side), but in order to adapt to the situation at hand; cognition has to take into account action's consequences.

### Conflict of interest statement

The authors declare that the research was conducted in the absence of any commercial or financial relationships that could be construed as a potential conflict of interest.
