# Supplementary figures and images for: When “good” is not always right: effect of the consequences of motor action on valence-space associations
Source: Front Psychol. 2015 Mar 5;6:237. doi: 10.3389/fpsyg.2015.00237 (PMC4350399; doi:10.3389/fpsyg.2015.00237)

Annexe 1. Animals used


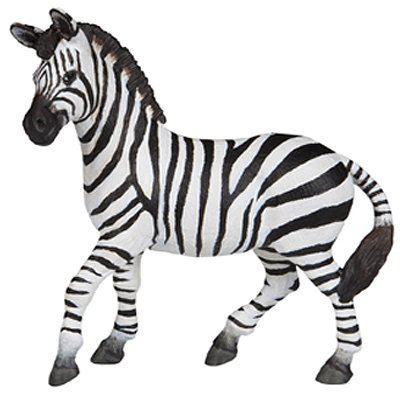


Good zebra


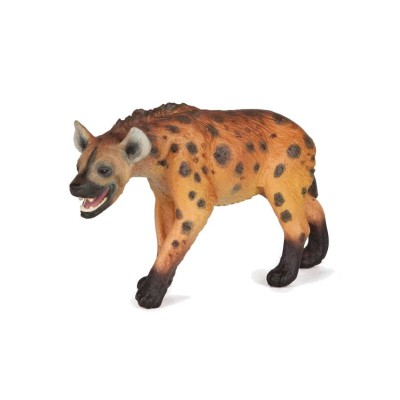


Bad hyena

Annexe 2. Experimental devices

Supplement: Supplementary file 1 [file DataSheet1.DOCX]
